# Amaranth Supplementation Improves Hepatic Lipid Dysmetabolism and Modulates Gut Microbiota in Mice Fed a High-Fat Diet

**DOI:** 10.3390/foods10061259

**Published:** 2021-06-01

**Authors:** Yongshou Yang, Rikako Fukui, Huijuan Jia, Hisanori Kato

**Affiliations:** Graduate School of Agricultural and Life Sciences, The University of Tokyo, 1-1-1 Yayoi, Bunkyo-ku, Tokyo 113-8657, Japan; yang.yongshou@mail.u-tokyo.ac.jp (Y.Y.); hansaallee26@gmail.com (R.F.)

**Keywords:** amaranth, high-fat diet, lipid metabolism, gut microbiota, cholesterol, triglycerides

## Abstract

Diet-induced obesity is often associated with gut microbiota dysbiosis, lipid metabolism disorders, and chronic inflammation. Consumption of the pseudocereal *Amaranthus mangostanus* has multiple nutritional benefits. We investigated the effects of dietary amaranth on lipid metabolism and gut microbiota in high-fat (HF) diet-fed mice. C57BL/6J mice were provided either a control diet, HF diet, or HF diet containing 10% amaranth powder (Ama) for 8 weeks. Ama supplementation significantly reduced the levels of triglycerides, total cholesterol, and phospholipids in the liver. Moreover, Ama supplementation downregulated the expression of lipogenesis-related genes including *Hmgcr*, *Fdt1*, and *Sgle* in the liver. The gut microbiota analysis showed that Ama supplementation reversed HF diet-induced reduction in bacterial diversity and richness. Additionally, beta diversity analysis of the inter-group variability in community structure showed a clear separation between the HF and Ama groups. Linear discriminant analysis effect size analysis revealed that 11 taxa were enriched in the Ama group, whereas 9 taxa were increased in the HF group. We found that family Porphyromonadaceae and unclassified S24-7 showed a strong positive and negative correlation with the lipid parameters, respectively. Taken together, these results indicated that dietary Ama may attenuate HF diet-induced deterioration of gut microbiota structure and hepatic lipid metabolism.

## 1. Introduction

The increasing prevalence of obesity is a growing epidemic health concern worldwide. Obesity is characterized by the gain of body weight, abnormal lipid metabolism, insulin resistance, gut microbiota dysbiosis, and chronic inflammation [1]. Insulin resistance in individuals with obesity results in an increased flux of free fatty acids that contribute to diabetic dyslipidemia, which is characterized by an increased level of triglycerides, a decrease in the level of high-density lipoprotein cholesterol, and an elevated level of low-density lipoprotein cholesterol [2]. Dyslipidemia is an important risk factor for cardiovascular disease development in individuals with diabetes. Among the approaches investigated for managing obesity, dietary interventions have been broadly considered owing to their safety, wide availability, and low cost. For instance, certain well-known natural products, including blueberries, green tea, red beans, and Chinese herbs, have been shown to exert preventive effects against obesity and related metabolic complications [3].

High-fat (HF) diet is a major factor that induces gut microbiota dysbiosis. Gut microbiota dysbiosis is associated not only with intestinal disorders but also with various other extra-intestinal diseases including metabolic disorders. For instance, previous studies have found that an elevated level of the endotoxin lipopolysaccharide (LPS) derived from intestinal Gram-negative bacteria, is associated with metabolic inflammation and insulin resistance in obese subjects [4]. Therefore, several efforts have been made to target gut microbiota for the treatment of metabolic disorders.

The pseudocereal *Amaranthus mangostanus*, which originates from the Andean region, is a dicotyledonous plant. Previous studies have reported that amaranth grains contain high levels of high-quality protein, minerals, and vitamins, as well as large amounts of other bioactive components with health benefits, including flavonoids, phytosterols, and polyphenols [5,6,7]. Owing to this composition, amaranth has been demonstrated to have antioxidant, anti-tumor, and anti-microbial activities, and preventive activity against cardiovascular diseases [6,8]. In addition, dietary amaranth leaves or seeds were reported to exert hypolipidemic effects [9,10]. However, few studies have examined the effects of amaranth consumption on gut microbiota, which has an impact on the development of several chronic diseases. Increasing studies have suggested an association between gut microflora and lipid metabolism; however, the association remains poorly understood. Therefore, we aimed to examine that how amaranth consumption affect the lipid metabolism and gut microbiome in HF diet-fed mice.

## 2. Materials and Methods

### 2.1. Animal Care and Diet

Seven-week-old male C57BL/6 J mice (Charles River Laboratories, Tokyo, Japan) were acclimatized for 7 d and then were randomly assigned to one of three groups according to the experimental diets: normal diet (Con: n = 6), HF diet (45% calorie from fat; HF: n = 8), and HF diet + 10% (*w*/*w*) amaranth powder (Ama: n = 8) (*Amaranthus mangostanus* used in this study was obtained from Peru) for 8 weeks. The mice had ad libitum access to either the experimental diets or fresh water. The diet compositions are shown in Table S1. The animal experiment was approved by the Animal Care and Use Committee of the University of Tokyo.

### 2.2. Sample Collection and Biochemical Assays

After treatment for 8 weeks, all mice were euthanized with pentobarbital sodium injection. Serum and the other major tissues were collected and stored at −80 °C for further analysis.

The extraction of lipids in liver was performed with chloroform-methanol (2:1, *v*/*v*) according to the Folch’s method. Subsequently, the concentrations of triglycerides (TG), total cholesterol (TC), high-density lipoprotein cholesterol (HDL), and phospholipids (PL) in liver and serum were determined using the kits LabAssay™ Triglyceride, LabAssay™ Cholesterol, HDL-Cholesterol-E, and LabAssay™ Phospholipids obtained from FUJIFILM Wako (Tokyo, Japan), respectively. All experiments were performed based on the manufacturer’s protocols.

### 2.3. Cecal Lactate and Short-Chain Fatty Acid (SCFA) Analysis

The concentrations of lactate and SCFAs present in cecal contents were measured by performing ion-exclusion high-performance liquid chromatography as described previously [11].

### 2.4. RNA Extraction and Real-Time PCR

Total RNA was extracted from liver tissues with the TRIzol^TM^ reagent (Ambion^®^ Life Technologies™, Foster City, CA, USA) and an RNA Isolation Kit (NucleoSpin RNA II; Macherey Nagel, Düren, German). cDNA was synthesized from extracted total RNA with the PrimeScript^TM^ RT Master Mix (Takara Bio, Tokyo, Japan) according to the manufacturer’s instructions. The primer sequences used in the study are described in Table S2.

### 2.5. Bacterial DNA Extraction and 16S rRNA Gene Sequencing

Total bacterial DNA in the cecal contents was extracted with QIAamp Stool Mini Kit (Qiagen, Hilden, Germany) according to the protocol. Subsequently, the extracted bacterial DNA was quantified via NanoDrop spectrometry. The V3–V4 region of the 16S rRNA gene was amplified with universal primers described in Table S2, followed by incorporating Illumina adapters and barcode sequences for further sequencing. Subsequently, the library size was confirmed using the Agilent 2100 Bioanalyzer (Agilent Technologies, Santa Clara, CA, USA), and all libraries were pooled in a single Illumina MiSeq run (MiSeq Reagent Kit V3; Illumina, San Diego, CA, USA) according to the manufacturer’s instructions.

### 2.6. Bioinformatics Analysis

Sequencing data were analyzed using QIIME2 (v. 2019.10) [12]. Following the pipeline, paired-end sequences were imported into QIIME2, and trimmed and combined using DADA2. The sequences were grouped into an amplicon sequence variant table based on 100% sequence similarity. Subsequently, alpha diversity and beta diversity were analyzed with QIIME2, and plotted using the R software (v.3.6.1). For taxonomic classification, Greengenes (13_8 99% OTUs) was utilized as a 16S rRNA gene database. The microbial functionality profiles were predicted with PICRUSt2 (v.2.1.4) to generate the Kyoto Encyclopedia of Genes and Genomes (KEGG) pathway data [13]. The predicted metagenomic data were aligned to the KEGG database, and the difference between groups was compared with STAMP (Welch’s *t*-test, Two-sided).

### 2.7. Data Analysis

Data are shown as the means ± standard error (SE). Statistical analysis was performed by one-way analysis of variance and Tukey’s post hoc test using the R software (v.3.6.1). Certain bacterial taxa data were evaluated via linear discriminant analysis effect size (LEfSe) analysis. The data separation in the principal coordinate analysis ordination of beta-diversity was conducted with the PERMANOVA permutation-based statistical test in vegan-R (“beta-group-significance” QIIME2 plugin). A value of *p* < 0.05 was considered statistically significant.

## 3. Results

### 3.1. Ama Supplementation Did Not Significantly Affect Body Composition in HF Diet-Fed Mice

**Figure 1:**
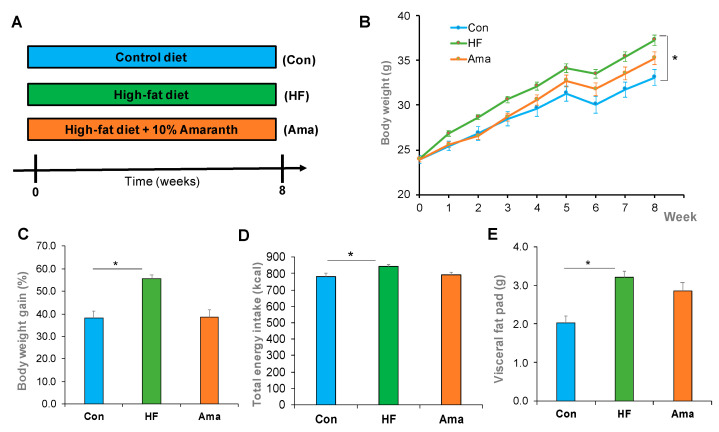
Effect of amaranth (Ama) supplementation on body composition in high-fat (HF)-fed mice. (**A**) mice were fed with normal diet (Con: n = 6), HF diet (HF: n = 8), or HF diet supplemented with 10% amaranth powder (Ama: n = 8) for 8 weeks. (**B**) During the treatment period, body weight (**B**) was monitored for each group. After 8-week treatment, body weight gain (**C**), total energy intake (**D**), and visceral fat pad weight (**E**) were calculated. Data are means ± SE. Statistical analysis was performed by one-way ANOVA and Tukey’s post hoc test. * *p* < 0.05.

During the 8-week experimental period, the total energy intake was significantly higher in the HF group than that in Con group (*p* < 0.05). The final body weight of mice significantly increased in the HF group, but not in the Ama group, compared with that of mice in the Con group (Figure 1B). The body weight gain, total energy intake, and the visceral fat pad weight were significantly increased in the HF group compared to that in the Con group. However, no statistical changes were observed in the Ama group compared to that in the other two groups (Figure 1C–E).

### 3.2. Ama Supplementation Modulated Lipid Metabolism in HF Diet-Fed Mice

**Figure 2:**
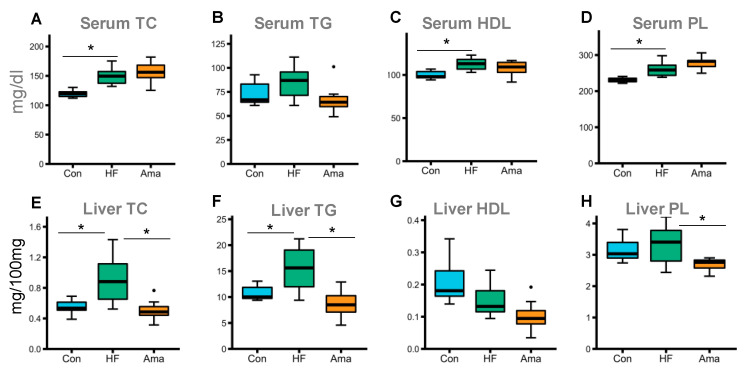
Modulatory effect of Ama on lipid parameters of serum and liver in HF-fed mice (Con: n = 6, HF and Ama: n = 8, respectively). After 8-week treatment, the levels of total cholesterol (TC) (**A** or **E**), triglycerides (TG) (**B** or **F**), high-density lipoprotein (HDL) (**C** or **G**), and phospholipids (PL) (**D** or **H**) in serum or liver were measured. Data are means ± SE. Statistical analysis was performed by ANOVA and Tukey’s post hoc test. * *p* < 0.05. The dots (●) in the boxplots are outliers.

Biochemical analysis demonstrated that the levels of TC, HDL, and PL in serum were significantly higher in the HF group compared to that in the Con group (Figure 2A,C,D). In the liver tissue, Ama supplementation reduced the TG, TC, and PL levels compared to those in the HF group (Figure 2E,F,H).

**Figure 3:**
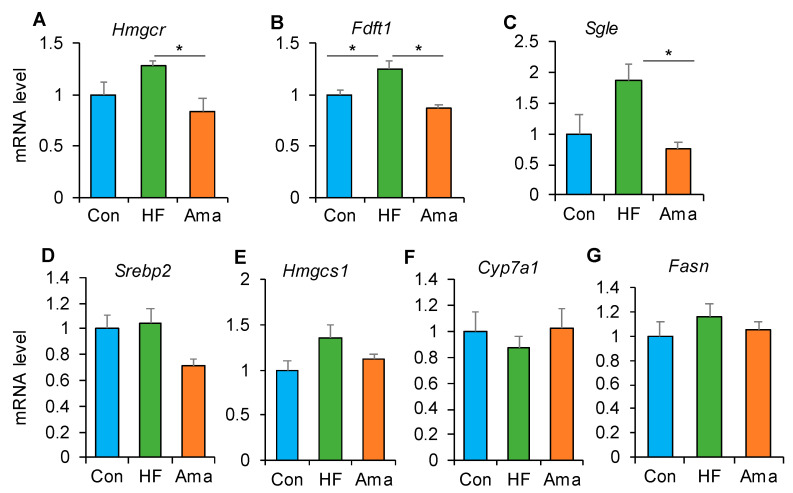
Ama modulates genes expression associated with lipid metabolism in liver (Con: n = 6, HF and Ama: n = 8, respectively). After 8-week treatment, genes expression related with cholesterol and triglyceride metabolism, such as *Hmgcr* (**A**), *Fdft1* (**B**), *Sgle* (**C**), *Srebp2* (**D**), *Hmgcs1* (**E**), *Cyp7a1* (**F**), and *Fasn* (**G**) in liver was examined using quantitative RT-PCR. Data are means ± SE. Statistical analysis was performed by ANOVA and Tukey’s post hoc test. * *p* < 0.05.

To investigate the molecular mechanism underlying the improvement in lipid profile by Ama supplementation, we analyzed the gene expression of lipid metabolism-related genes in the liver via RT-PCR analysis. Ama supplementation suppressed the gene expression of 3-hydroxy-3-methylglutaryl-CoA reductase (*Hmgcr*), farnesyl-diphosphate farnesyltransferase 1 (*Fdft*), and squalene monooxygenase (*Sgle1*), which are involved in cholesterol metabolism (Figure 3A–C).

However, the expression of other genes related to cholesterol and TG metabolism, including sterol regulatory element-binding protein 2 (*Srebp2*), 3-hydroxy-3-methylglutaryl-CoA synthase 1 (*Hmgcs1*)*,* cholesterol 7 alpha-hydroxylase (*Cyp7a1*), and fatty acid synthase (*Fasn*)*,* was not affected by Ama treatment (Figure 3D–G).

### 3.3. Ama Supplementation Modulated the Gut Microbiota in HF Diet-Fed Mice

**Figure 4:**
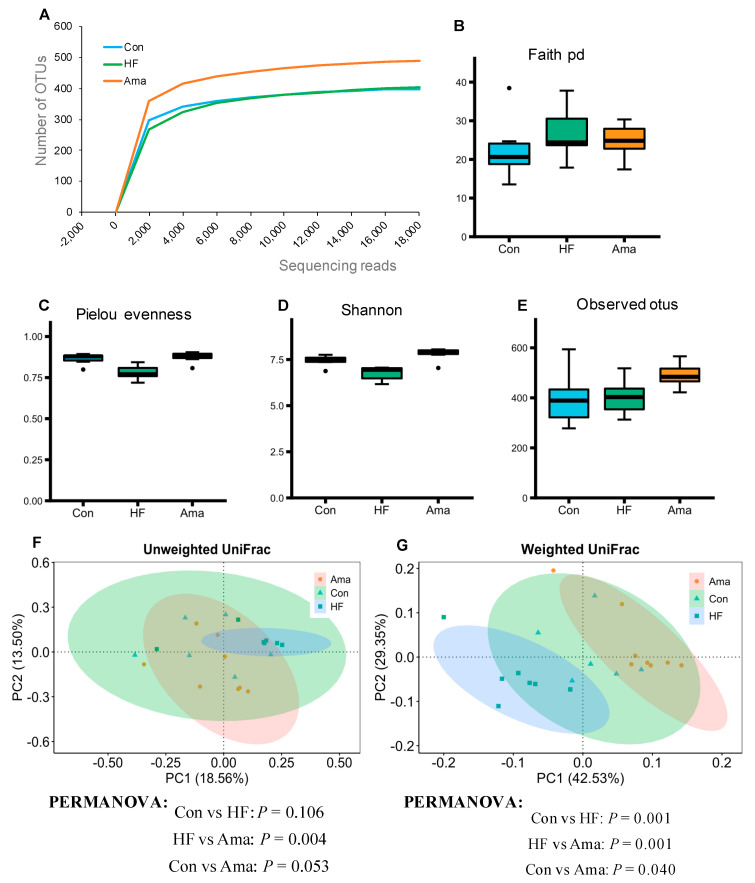
Effects of Ama on alpha- and beta-diversity of gut microbiota in HF-fed mice (Con: n = 6, HF and Ama: n = 8, respectively). After an 8-week treatment, bacterial DNA in cecal contents was collect for 16S rRNA gene sequence. (**A**) Rarefaction curves for the comparison of the microbial communities in the cecal contents. Diversity and evenness within sample were measured by (**B**) Faith’s phylogenetic diversity (faith pd); (**C**) Pielou’s evenness; (**D**) Shannon index; (**E**) Observed otus. The principal component analysis (PCoA) of Unweighted (**F**) and weighted UniFrac (**G**) and PERMANOVA analysis were performed to compare the gut microbiome profiles among the experimental groups. Statistical analysis was performed by ANOVA and Tukey’s post hoc test. * *p* < 0.05. The dots (●) in the boxplots are outliers.

For analyzing the microbiota, a total of 935,207 high-quality reads were processed by the QIIME2 filter. Rarefaction curves (Figure 4A) indicated that the number of OTUs tended to be stable when the reads number exceeded 4000. The alpha diversity analysis showed that the Faith PD, which is the phylogenetic diversity of gut microbiota, and Observed otus were not affected among the three groups (Figure 4B,E). However, the other two alpha diversity indices, Shannon and Pielous_evenness, showed that the Ama group presented a higher diversity and evenness of gut microbiota than those in the HF group (Figure 4C,D). The beta diversity (unweighted and weighted UniFrac distances) was analyzed to compare the overall structure of gut microbiota in the total samples (Figure 4F,G). Weighted UniFrac analysis indicated a distinct compositional difference between the Con and HF groups. Moreover, both unweighted and weighted UniFrac distance analysis revealed a distinct compositional difference between the HF and Ama groups.

**Figure 5:**
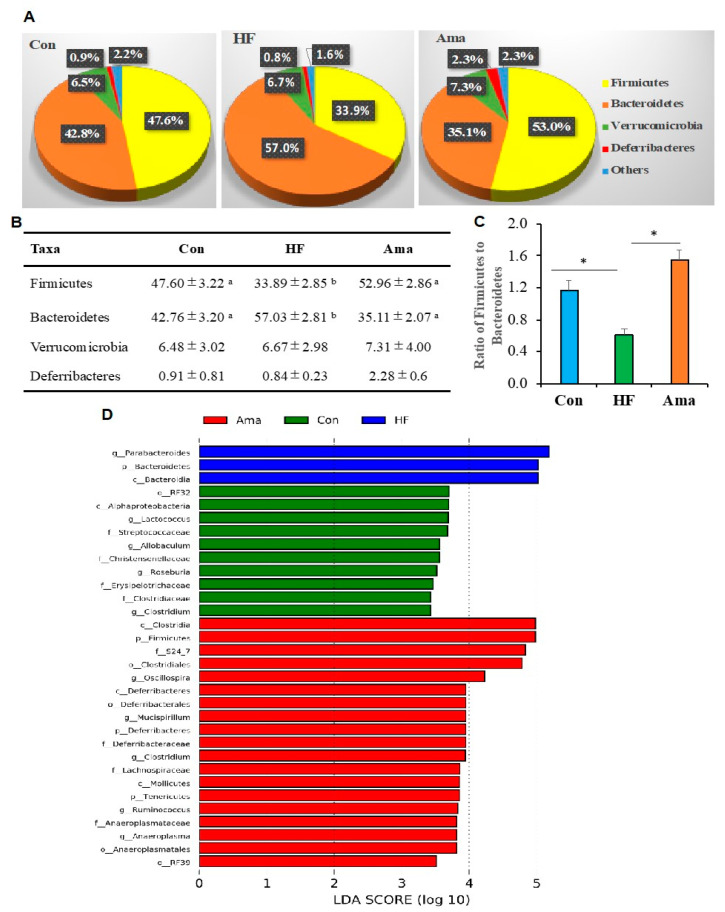
Effects of Ama on gut microbiota taxa composition in HF-fed mice (Con: n = 6, HF and Ama: n = 8, respectively). Relative abundance of phyla in these experimental groups is showed in pie graphs (**A**) and table (**B**). (**C**) Display of the ratio of Firmicutes to Bacteroidetes in all groups. (**D**) LEfSe analysis was performed to compare the different taxa among the three groups. * (**C**) and different letters (**B**) indicate significant difference at *p* < 0.05 by one-way ANOVA and Tukey’s test. For LEfSe analysis, the two-tailed nonparametric Kruskal–Wallis test was performed to evaluate the significance of differences in taxa.

Analysis of the taxonomic composition showed that all groups contained the four predominant phyla Bacteroidetes, Firmicutes, Verrucomicrobia, and Deferribacteres (Figure 5A,B). The level of Firmicutes was significantly higher whereas the level of Bacteroidetes was lower in the HF group compared to those in the Con and Ama groups. The ratio of the abundance of Firmicutes to Bacteroidetes in the HF group was statistically lower than that in the other two groups (Figure 5C).

LefSe analysis was conducted to compare the different taxa among the three groups. The abundance of 32 bacterial taxa were found to be altered among the experimental groups (Figure 5D). These taxa belonged to the phylum Bacteroidetes and genus *Parabacteroides* were present in higher proportions in the HF group than in the Con and Ama groups. The taxa that belonged to the class Alphaproteobacteria, and families Streptococcaceae*,* and Erysipelotrichaceae were present in higher proportions in the Con group than in the other groups. The taxa belonging to the phyla Firmicutes, Deferribacteres, and Tenericutes were distributed in higher proportions in the Ama group than in the other groups.

**Figure 6:**
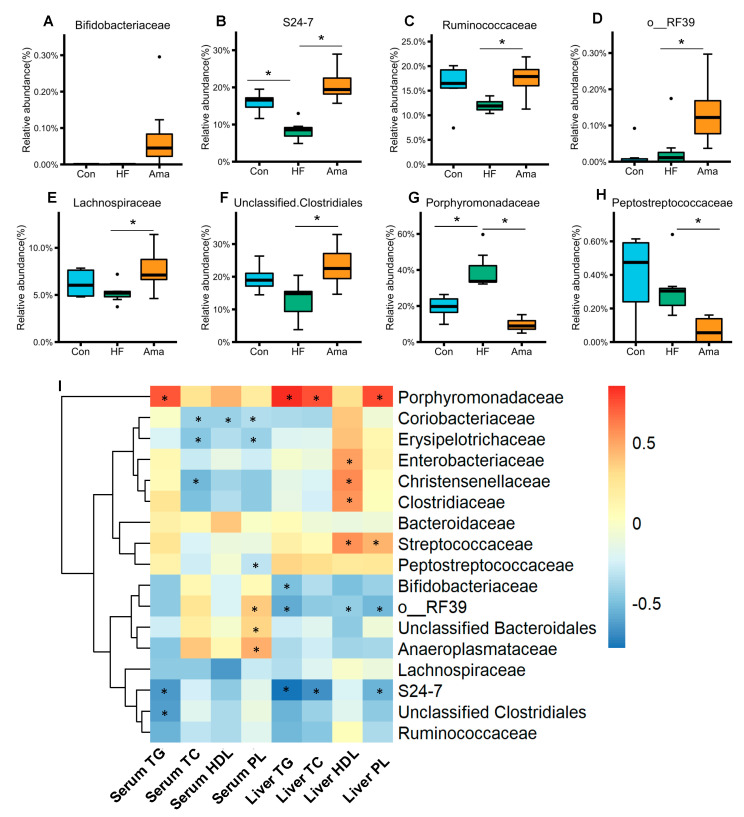
Effects of Ama on gut microbiota composition at the family level in HF-fed mice (**A**–**H**, Con: n = 6, HF and Ama: n = 8, respectively). (**I**) Pearson correlation between lipid parameters and relative abundance of gut bacteria at the family level. Statistical analysis in (**A**–**H**) was performed by one-way ANOVA and Tukey’s post hoc test. * *p* < 0.05. The dots (●) in the boxplots are outliers.

At the family level, the abundance of Bifidobacteriaceae tended to be higher in the Ama group than in HF group (*p* = 0.055, Figure 6A). The commensal bacteria including unclassified S24-7, Ruminococcaceae, unclassified Clostridiales, Lachnospiraceae, and unclassified RF-39 were significantly enriched in the Ama group compared to those in the HF group (Figure 6B–F). The abundance of Porphyromonadaceae and Peptostreptococcaceae (Figure 6G,H) was lower in the Ama group than in the HF group. Collectively, these results showed that Ama supplementation modulated the gut microbiota of HF diet-fed mice, resulting in a microbiota composition similar to that in Con mice.

The concentrations of SCFAs in the cecal contents, such as acetate, propionate, butyrate, and isobutyrate, were not affected by Ama supplementation (Figure S1). However, the level of lactate in the cecal contents was significantly higher in the HF group than in the other two groups.

### 3.4. Correlation between Gut Microbiota and Lipid Parameters

To further understand the relationship between specific bacterial taxa and lipid parameters, we generated a Pearson correlation heatmap by selecting bacteria at the family level and lipid parameters of the serum and liver (Figure 6I). A strong positive correlation was observed between Porphyromonadaceae and the levels of serum TG, liver TG, TC, and PL, respectively. In contrast, the negative correlations were found between unclassified S24-7 and these lipid parameters. Additionally, the negative correlations were observed between unclassified RF39 and the levels of TG, HDL, and PL in the liver, respectively.

### 3.5. Functional Analysis

**Figure 7:**
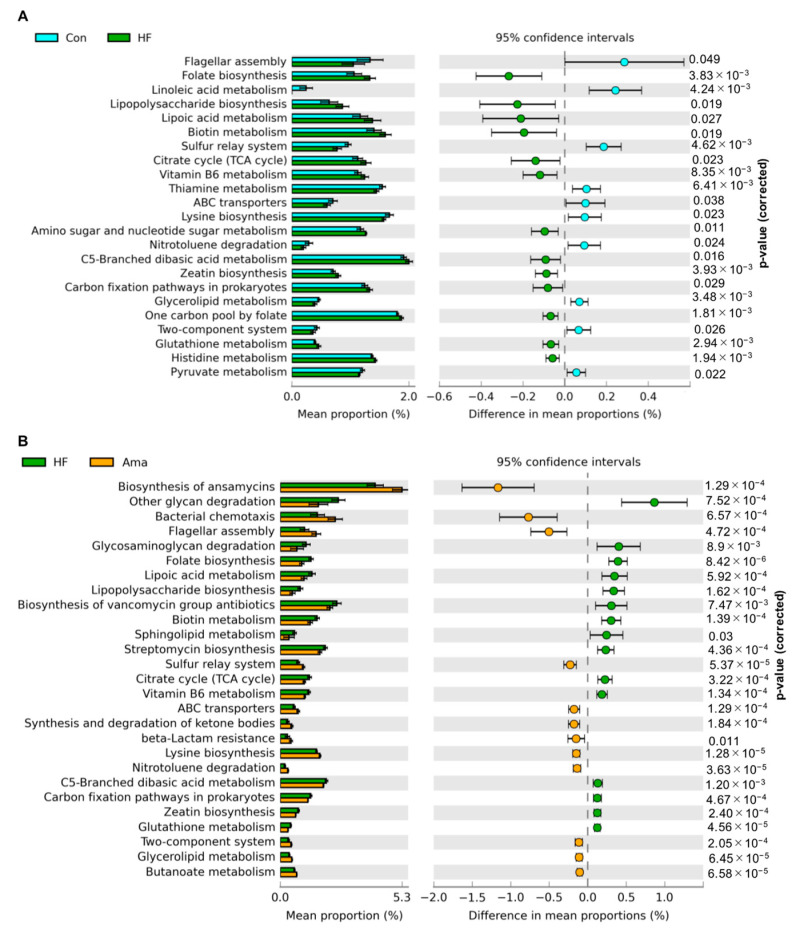
Prediction of bacterial genes functions to make inferences from KEGG annotated databases using PICRUSt analysis (Con: n = 6, HF and Ama: n = 8, respectively). (**A**) Collectively, 27 KEGG pathways were statistically altered in the HF group compared to that in Con group; (**B**) Totally, 16 KEGG pathways were reversed by Ama treatment. The mean proportion of each pathway is displayed in left Bar graphs.

To gain a better understanding of the changes in the gut microbiota after Ama supplementation, we performed PICRUSt analysis to predict the bacterial genes functional profiles in the treatment groups according to the composition of the gut microbiota. A total of 23 KEGG pathways associated with the gut microbiota were largely altered in HF group compared to those in the Con group (Figure 7); they included bacterial motility (flagellar assembly), energy metabolism (citrate cycle), lipid metabolism (lipoic acid metabolism), and inflammation process (LPS biosynthesis), among others. Compared with the HF group, Ama supplementation significantly influenced 27 KEGG pathways of the gut microbiota. Among them, Ama supplementation reversed 16 KEGG pathways that were affected by the HF diet, including inflammation process, bacterial motility, energy, and vitamin metabolism, among others.

## 4. Discussion

The pseudocereal amaranth contains various bioactive components that confer multiple health benefits and is thus referred to as a “superfood” [10,14]. In this study, we demonstrated that Ama supplementation attenuated HF diet-induced hepatic lipid dysmetabolism and modulated the composition of gut microbiota.

Generally, the long-term intake of western-style foods (containing high fat and high sugar) may be related with the development of metabolic disorders including hyperglycemia, dyslipidemia, obesity, glucose intolerance, and dysbiosis of gut microbiota. Herein, mice were given a HF diet for eight weeks, thereby inducing the development of metabolic disorders (Figure 1). Subsequent analyses showed that Ama supplementation significantly reduced TG, TC, and PL levels in the liver. Ama supplementation suppressed lipogenesis-related gene expression in HF diet-induced mice. Therefore, these data indicated that the alleviation of HF diet-induced hepatic lipid dysmetabolism via Ama supplementation might have occurred via the suppression of cholesterol synthesis in the liver. Several studies have reported that the intake of polyphenol- and dietary fiber-rich foods may alleviate metabolic syndromes by improving lipid metabolism [15,16,17]. Amaranth has been shown to contain large amounts of bioactive components such as polyphenols and dietary fibers [10,14]. Therefore, these components are highly likely to be associated with the observed effects of amaranth intake on the improvement of hepatic lipid dysmetabolism in HF diet-induced mice in this study.

Previous studies have demonstrated the consequences of alteration in the gut microbiota in HF diet-induced obesity and metabolic disorders [1]. Therefore, we investigated the effects of Ama supplementation on the composition of gut microbiota with 16S rRNA gene sequencing. A previous work found that the ratio of Firmicutes/Bacteroidetes (F/B) is increased in obese subjects and may be associated with a higher energy absorption from food and elevated low-grade inflammation [18]. However, contradictions regarding the alteration of F/B ratios in obesity subjects have been observed. For instance, reduced F/B ratios have been reported in obesity mice or humans in other studies [19,20]. In current study, the F/B ratio was decreased in HF group but elevated by Ama treatment. A previous study reported that a species of the *Bacteroides* genus, *Bacteroides thetaiotaomicron,* can increase fat storage in the host by downregulating the fasting-induced adipocyte factor (*Fiaf*) gene to coordinate higher lipogenesis in liver with increased lipoprotein lipase activity in mouse adipocytes [21].

In general, the consumption of prebiotics promotes the enrichment of beneficial gut bacteria, such as *Bifidobacterium* and *Lactobacillus* [22]. In our study, the abundance of the family Bifidobacteriaceae tended to be higher by Ama treatment. This result is consistent with the previous findings that Ama promotes the growth of probiotic bacteria in vitro, including *Bifidobacterium* [23]. Additionally, supplementation of *Bifidobacterium pseudolongum* significantly decreases the plasma TG levels in obese mice [24]. The current study found a strong negative correlation between the abundance of Bifidobacteriaceae and liver TG concentration. The potential regulatory role of Bifidobacteriaceae species in lipid metabolism should be investigated in future studies. Several species belong to the family Porphyromonadaceae are a part of indigenous gut bacteria of mammals; however, certain species are associated with various infections in human and animal. A study found a relatively higher abundance of Porphyromonadaceae in patients with colorectal cancer [25]. Our study confirmed that the abundance of Porphyromonadaceae was enriched by HF diet but was reduced by Ama supplementation. Furthermore, Pearson correlation analysis indicated a strong positive correlation between Porphyromonadaceae and the levels of serum TG, liver TG, TC, and PL, thereby suggesting an important role of such bacteria in lipid metabolism. Further studies are needed to elucidate the detailed roles of Porphyromonadaceae in host-lipid metabolism. Ama supplementation also significantly increased the level of butyrate-producing bacteria, such as Lachnospiraceae and Ruminococcaceae, which are normally considered beneficial intestinal bacteria. However, the concentration of butyrate was not affected by Ama treatment, suggesting that other factors may influence butyrate metabolism in the intestine.

Based on the gut microbiota composition, the PICRUSt analysis predicted that the Ama-modulated gut microbiota presented a higher bacterial motility, and lower lipid metabolism and carbohydrate metabolism than those of the other groups. Particularly, Ama-fed mice showed a reduced retardation in the activity of certain pathways that were previously shown to be associated with obesity and systemic inflammation, including LPS biosynthesis and sphingolipid metabolism [4,26]. Further studies are needed to examine the concentration of systemic LPS and other inflammatory markers after Ama supplementation.

There are some limitations should be pointed out in the current study. This study demonstrated the ameliorative effects of Ama supplementation on HF-induced hepatic lipid dysmetabolism and dysbiosis of gut microbiota but did not provide the underlying mechanism. The current research was an obesity prevention study that Ama was administered to lean mice starting when those mice were fed a HF diet, which limits its relevance to human populations. Further studies are necessary to elucidate these issues.

In summary, our results showed that Ama supplementation ameliorated hepatic lipid dysmetabolism by reducing the levels of liver TG, TC, and PL, and inhibiting the expression of lipogenesis-related genes in liver. Further, Ama supplementation improved the gut microbial dysbiosis in HF diet-fed mice, as indicated by the higher diversity and richness, and the elevated abundance of beneficial gut bacteria (Bifidobacteriaceae and butyrate-producing bacteria). These results suggest that amaranth may be used as a prebiotic for improving lipid metabolism and gut health by modulating gut microbiota.

## Data Availability

The 16S rRNA gene sequence data have been deposited in the DDBJ database (http://getentry.ddbj.nig.ac.jp/ (accessed on 12 April 2021)) under accession number PRJDB11515.

## Supplementary Materials

The following are available online at https://www.mdpi.com/article/10.3390/foods10061259/s1, Table S1: Composition of experimental diets, Table S2: The list of primers used in the study, Figure S1: Effect of Ama on organic acids in cecal contents of mice.
