# Supplementary material for: Amaranth Supplementation Improves Hepatic Lipid Dysmetabolism and Modulates Gut Microbiota in Mice Fed a High-Fat Diet
Source: Foods. 2021 Jun 1;10(6):1259. doi: 10.3390/foods10061259 (PMC8229566; doi:10.3390/foods10061259)
Supplement: Supplementary file 1 [file foods-10-01259-s001.zip › foods-1201164-supplementary.pdf]

Table S1. Composition of experimental diets

Ingredient (%)	Con	HF	Ama
Casein	25.60	25.60	24.32
Cystine	0.36	0.36	0.36
Maltodextrin	6.00	6.00	6.00
Corn starch	46.25	30.75	22.67
Sucrose	5.50	5.50	5.50
Soybean oil	7.00	2.00	1.36
Lard	-	20.50	20.50
Cellulose	4.61	4.61	4.61
AIN-93G Mineral mixture	3.50	3.50	3.50
AIN-93 Vitamin mixture	1.00	1.00	1.00
Calcium carbonate	0.18	0.18	0.18
Amaranth	-	-	10.00
Total calory (kcal/100g)	397.8	475.3	469.4
Protein (kcal/100g)	104	104	104
Carbohydrate (kcal/100g)	231	169	163
Fat (kcal/100g)	63.0	202.5	202.5
Dietary fiber (g/100g)	4.61	4.61	5.25

Table S2. The list of primers used in the study

Gene	Full name		Primer sequence (5'-3')
Universal	universal bacterial primers	Forward	CCTACGGGNGGCWGCAG
		Reverse	GACTACHVGGGTATCTAATCC
Fasn	fatty acid synthase	Forward	TTGTCGTCTGCCTCCAGAGC
		Reverse	GACCATGTCCACACCACCAA
Cyp7a1	cholesterol 7a- hydroxylase	Forward	GGGGATTGCTGTGGTAGTGAG
		Reverse	GCACAGCCCAGGTATGGAA
Srebp2	sterol regulatory element binding protein 2	Forward	AAGCTGGGCGATGGATG
		Reverse	CCACTTGATTGCTGACAAACTG
Hmgcs1	3-hydroxy-3-methylglutaryl-CoA synthase 1	Forward	ATGAACCATCAGTGAGAGGAAGG
		Reverse	ACGGAGACCTGGCCAAAA
Hmgcr	3-hydroxy-3-methylglutaryl-CoA reductase	Forward	CAGTACAGTCGTCATTCATTTCCTC
		Reverse	AACTTTGCTAATGCACTCGCTCT
Fdft1	farnesyl-diphosphate farnesyltransferase 1	Forward	AATCAGACCAGTCGCAGCTTT
		Reverse	CAGTGGGATCTTCTTCTCCACAC
Sqle	squalene monooxygenase	Forward	GCTTTGCCTCTTATTGGCTTCTT
		Reverse	GCTGCTCCTGTTAATGTCGTTTC
Actb	Actin beta	Forward	GACGGCCAGGTCATCACTAT
		Reverse	CTTCTGCATCCTGTCAGCAA

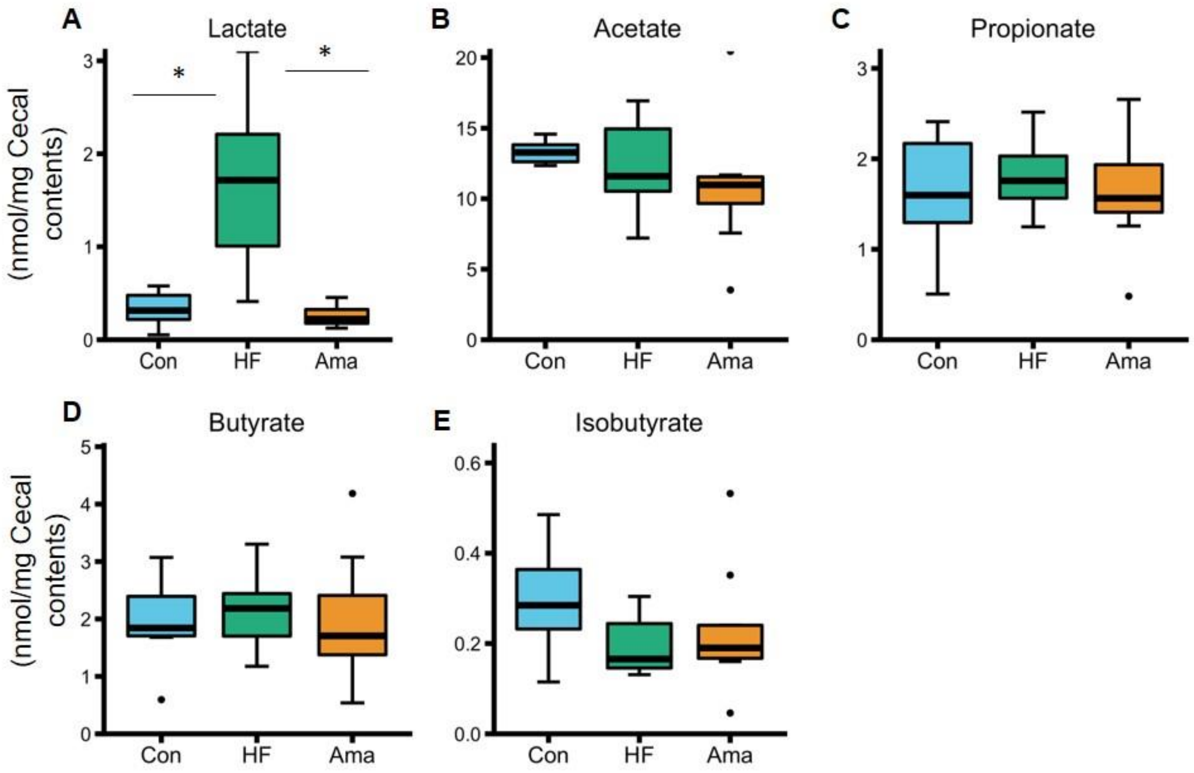

Figure S1. Effect of Ama on organic acids in cecal contents of mice. After 8-week treatment, cecal levels of lactate (A), acetate (B), propionate (C), butyrate (D), and isobutyrate (E) were analyzed. Statistical analysis was performed by one-way analysis of variance and Tukey's post hoc test. *P <0.05.
